# Misplacement of an internal jugular hemodialysis catheter due to stenosis caused by previous short-term catheterization

**DOI:** 10.4103/0972-5229.53118

**Published:** 2009

**Authors:** Prakash K. Dubey

**Affiliations:** **From:** Department of Anesthesiology and Critical Care Medicine, Indira Gandhi Institute of Medical Sciences, Patna, India

Dual lumen hemodialysis catheters are usually inserted via the right internal jugular vein (IJV). Subclavian vein (SCV) is generally avoided to prevent thrombosis or narrowing.[1] The site of stenosis caused by SCV catheter is usually near the junction of the SCV and IJV.[2]

A 30-year-old female, suffering from endstage renal disease, was in the operating room for insertion of a hemodialysis catheter. She had undergone right IJV catheterization about 45 days back. The previous catheter had remained *in situ* for about 15 days. The exact cause of catheter removal was not documented. As she had no clinical signs or symptoms in the upper limb or the neck suggesting any complication, it was decided to cannulate the right IJV again.

**Figure 1:**
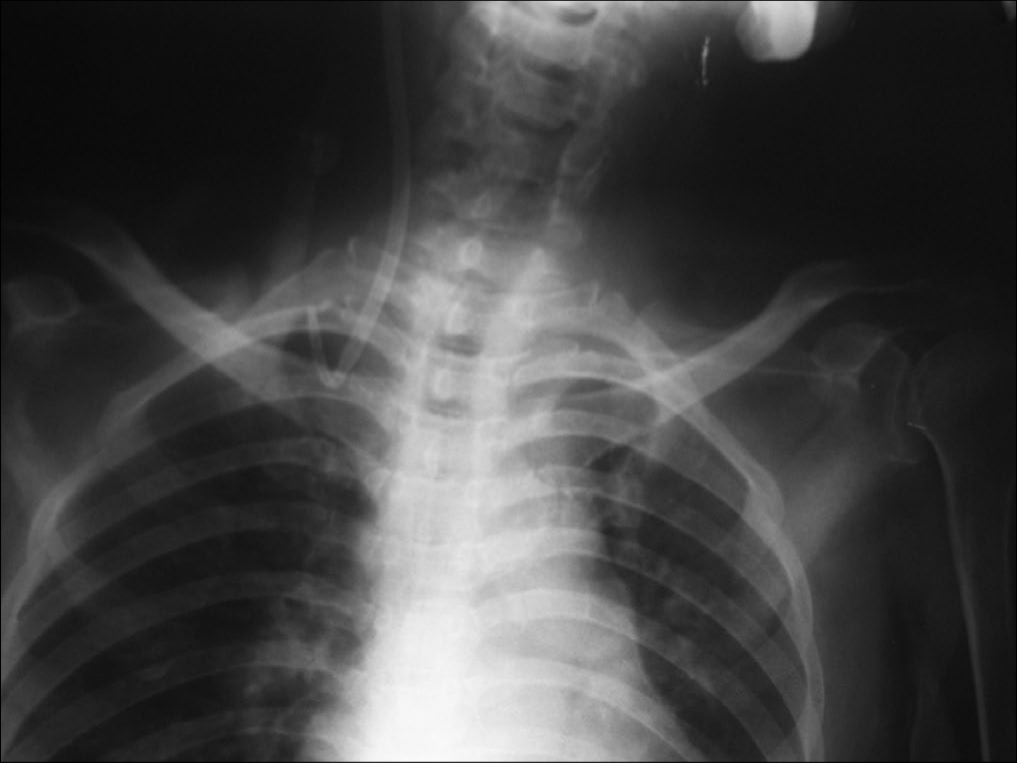
Chest X-ray showing the misplacement of right internal jugular hemodialysis catheter

After instituting monitoring with lead II electrocardiogram, noninvasive blood pressure, and a pulse oximeter, a 12.5 Fr hemodialysis catheter (Soft-cell^®^ PC kit, Bard Access Systems, Utah, USA) was placed uneventfully via the right IJV under local anesthesia with full aseptic precautions. Free aspiration of blood via both the lumens was attained through a 5-ml syringe. A chest radiograph was obtained postprocedure and haemodialysis was started without inspecting the X-ray film. However, a satisfactory blood flow through the hemodialysis machine could not be obtained. Gentle manipulation of the catheter and change in patient position made no difference to the flow. Meanwhile, the X-ray film revealed catheter misplacement into the ipsilateral SCV [Figure 1].

The patient was transferred back to the operating room and the catheter was partially withdrawn over a guidewire using an image intensifier. At that time it became difficult to pass the guidewire beyond the junction of right IJV and SCV. Everytime an attempt was made the guidewire was deflected into the SCV. After gentle manipulations, the guidewire entered medially into the brachiocephalic vein but the catheter could not be advanced over it. The guidewire along with the catheter was partially withdrawn and the catheter was repositioned keeping its tip proximal to the site of the suspected stenosis. The catheter was used for hemodialysis without any complication.

Postcatheterization stenosis following long-term right IJV catheterization is not unknown. The prevalence of stenosis following SCV cannulation has been found to be 40–50% and stenosis resulting from cannulation of IJV 0–10%.[3] Anatomic narrowing can be present without clinical consequences,[4] as was seen in this patient. Stenosis has been defined as 50% or greater diameter reduction in the vein with or without collateral vessels.

This patient had a previous catheter *in situ* for only 15 days, so it was thought that a reinsertion would be safe on the same side. But this was enough to cause a stenosis at the site described. Probably the lateral pressure caused by the catheter movement on the vein wall at the junction of IJV and SCV led to this stenosis.

This suspected stenosis deflected the guidewire laterally into the SCV. The lower tapered part of the catheter entered the SCV following the guidewire in a lateral direction. Blood could be aspirated freely through a syringe as probably the kin k was not enough to occlude blood flow at low pressure. Fortunately there was no trauma leading to further complications.

It has been recommended to use ultrasound examination prior to IJV catheterization, especially in patients with previous temporary or tunneled catheters.[5] The authors found IJV stenosis in five out of 100 such patients. Significantly, catheter could be placed through four of these five stenotic lesions. It is known that ultrasound cannot visualize and directly evaluate the brachiocephalic vein and superior vena cava, and Doppler waveform analysis should be added to detect any stenosis.[5] Such a device could have suspected a stenosis in this patient even in the absence of collaterals.
